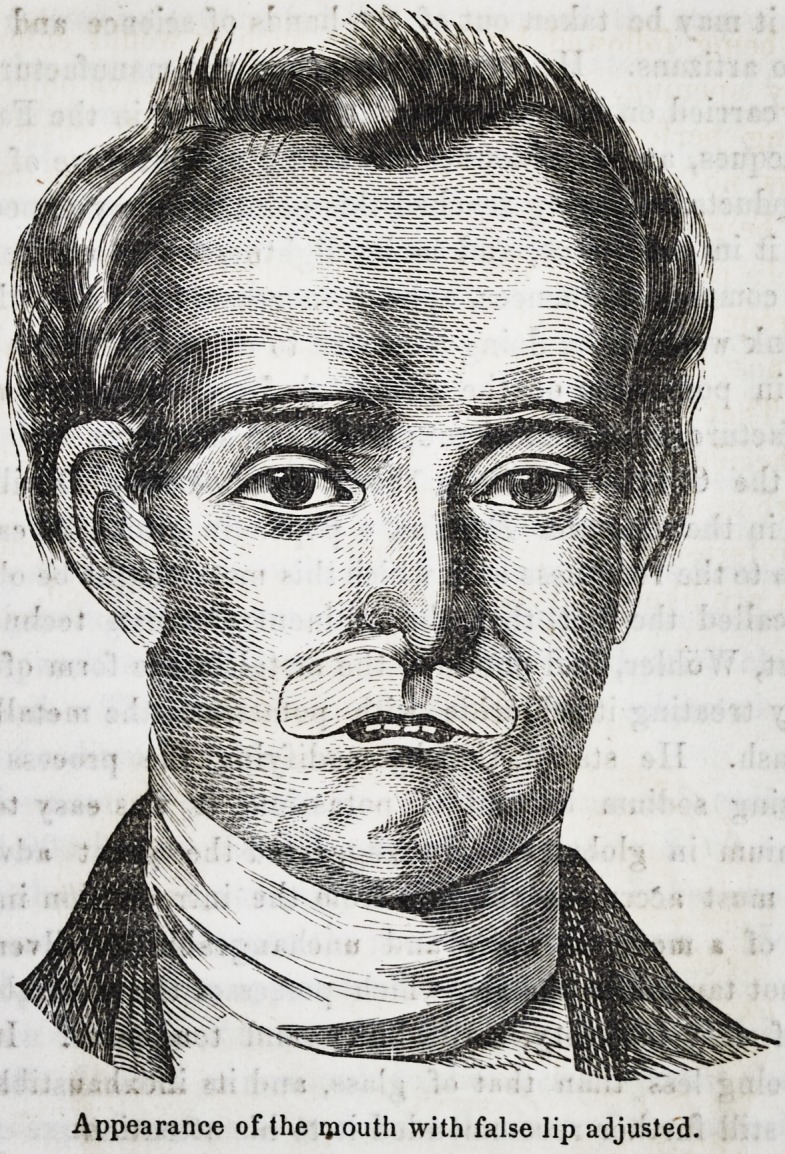# A Case of Destruction of the Entire Palate, Successfully Relieved by Mechanical Means

**Published:** 1857-04

**Authors:** Edwin Sercombe


ARTICLE II.

*A Case of Destruction of the Entire Palate, Successfully Relieved by Mechanical Means.* By Edwin Sercombe, M. R. C. S. Communicated by Benjamin Travers, F. R. S.
Received Jan. 19th, 1856.—Read Jan. 23d, 1856.

In one of the earliest volumes of the Transactions of this Society, the President and Council invite "solitary cases, when possessed of peculiar novelty and interest, as deemed by the Society very appropriate objects of communication." Upon this invitation the following case is brought forward, with a hope that though, in some respects, it may not be regarded as a solitary one, it possesses sufficient novelty and interest to commend it to the attention of this Society.

It is intended to omit, on the present occasion, all reference to the early history of this case, and not even to venture an opinion as to what the disease really was, which worked with such destructive energy on the unfortunate individual who has supplied a text for the following account; because, interesting as such an inquiry might be, it would be beside the point which it is the object of this paper to develop, and which it is of more than ordinary importance to establish, viz. that it is within our reach to restore, by mechanical contrivance, one or more portions of the face, which from disease or injury may have been destroyed.

The following case offered difficulties not likely to be often surpassed, which were increased by an insuperable objection, on the part of the patient and his family, on account of his profession, to the adoption of a beard and moustache, by which the deformity might have been much more easily concealed.

Fourteen years of suffering—always severe, often terrible,—terminated in a rest, which, to the subject of our present remarks, was almost more calamitous than the agony he had endured; for it was a rest enforced by a disfigurement so great, as to render him altogether unfit to occupy a post for which he was pre-eminently fitted, and to confine him almost within the limits of his immediate family.

Drawing No. 1, represents the face as injured by disease.

Drawing No. 2, represents the face with an artificial upper lip adapted to it.

The external disfigurement is, however, but imperfectly represented by these drawings, for the animation of his eye lent a light, as it were, to bring out the hideousness of a face distorted, contracted and discolored by recent disease, but, with this modification, they are perfectly correct.

The work of destruction may be described in few words: the soft parts in the fauces were attacked with the most virulent ulcerations, and sloughed away in masses; our patient was rapidly reduced from thirteen stone to below ten; bark and iodine were administered, and he as rapidly recovered weight, gaining twelve pounds in twelve days. The disease, however, was not stayed, the upper front teeth became black, and several were removed; the gums, instead of healing, ulcerated, and portions of the alveolar processes exfoliated. In this way the upper incisors, canines, and bicuspids were lost. The ulceration now crept onwards to the face, the nose was attacked and partially destroyed, the lips completely so; indeed the whole of the face, below a straight line drawn from one meatus auditorius externus over the lower margin of the nasal bones to the other meatus, appears to have been subjected to the same destructive process, for it is intersected in every direction with cicatrices. The intermaxillary and palate bones exfoliated in large pieces; hemorrhage from the palatine arteries, and other smaller branches, lent its terrors to the sufferer, who says, in a note of his case about this time, "very extensive loss of bone of palate and upper jaw, with bleeding from the inside of nose and mouth—excessive distress." The vomer had disappeared. Considerable portions of the inferior maxillary had exfoliated, and all the teeth, save a canine and two incisors, represented in Drawing No. 2, had disappeared.

An external examination of the face displayed, as is shown by the Drawing No. 1, partial destruction of the alæ nasi, especially on the right side and complete loss of both lips, as the band stretching across, representing the upper lip, could scarcely be called a lip—it was a firm, unyielding mass of abnormal tissue; thus the cavity of the mouth was at all times exposed through an irregularly oblong opening, represented in the drawing, on the lower border of which stood the only three teeth found in the lower jaw when I first saw the patient; and evidences of former terrible ulcerations appeared in cicatrices which mapped the face in every direction below the line mentioned above. The contraction of the face during the healing of the ulcerations, had not been less than an inch and a half, measuring from the line where the hair ceased to grow on the forehead, to the lower border of the inferior maxilla; and as the shortening of the face was entirely within the lines across the middle of the face above, and the lower border of the inferior jaw below, the face might be said to be very much out of drawing; add to this, that the greater part of the hole leading into the mouth was to the right of the median line, and we have then a tolerably correct idea of the unsightly appearance of the countenance. In consequence of this contraction, and the rigid condition of the cicatrices, the lower jaw could not be depressed more than the eighth of an inch. The inability to depress it to a greater extent, depended, not upon any injury of the articulation, but upon the unyielding character of the abnormal tissues of the face; for when a free incision was made from the left extremity of the hole towards the left ear, which separated some of the most firm of the bands, considerably increased motion was at once obtained.

On looking into the ori-nasal cavity (for the two were thrown completely into one) which could be as conveniently done through the right nostril as through the opening below it, it was found to be triangular in shape, with a blunted apex, formed by the lower surface of the cribriform plate of the ethmoid (the central lamella was gone,) the base formed by the tongue, and the sides by the superior and middle turbinated bones, lower down by the nasal wall of the antrum of Highmore, on one side by three molar teeth, and on the other by two.

The models and diagrams render any further description of the destruction of parts within and about the mouth unnecessary; we will therefore proceed now to describe the apparatus made. The utmost that was hoped for, when the case was first undertaken, was to render our patient intelligible to his own immediate family and friends; for this purpose it was determined to construct, if possible, some sort of obturator. At this stage of the case, nothing further was contemplated, nor was it until the obturator had been worn some time, that any attempt at making lips was undertaken. The different steps of the task shall be briefly described.

The first step was to remove the three teeth which stood at the entrance of the mouth, for, until this was done, it was impossible to introduce even a single finger; but, although considerable space was thus gained, sufficient in fact to allow two fingers to be passed together easily into the mouth, yet the want of room to manipulate was so great that I could not refrain from suggesting that an incision be made; and after consultation with Mr. Travers, (under whose care our patient had been for the last three or four years, until the completion of the cicatrizing process and the restoration of his physical powers,) it was determined to make one of an inch and a half or two inches long, from the left corner of the mouth towards the left ear, and to endeavor to stitch the mucous membrane of the mouth to the skin along the line of incision, so as, if possible, to induce union by the first intention, in which case it was hoped that a permanent opening would be obtained sufficiently large to admit of the introduction of an obturator of the ordinary description. This operation was performed by Mr. Holmes, late house-surgeon of St. George's Hospital; but from the altered state and unyielding consistence of the parts around the opening, it was found impossible to bring the mucous membrane sufficiently close to the skin, union by the first intention did not ensue, and although the incision healed kindly, the contraction was so great that when it was perfectly healed, it was found that no increase of room had been gained. It was now obvious that no ordinary mode of treating the case could be employed; for the first step, whether for making artificial teeth or an obturator, is to get a perfect model of the whole jaw, either upper or lower, as the case may be, in softened beeswax, from which a plaster of Paris model is obtained; but in the present case, it was absolutely impossible to get in one piece a model of the whole of even one side of what remained of the upper jaw, much less of both; therefore, at the very outset, some new plan had to be devised by which a model could be obtained. The following was the plan adopted: a tray of metal, shaped somewhat like the handle of a spoon, was armed with softened wax, introduced into the mouth and pressed against the sides of the teeth of one side, together with as much of the surface of the bone above them as it would cover, it was then carefully removed, bringing on its surface a correct impression of the parts with which it had been brought into contact; the same operation was performed on the other side, and plaster of paris models were obtained from them; but as the impression thus obtained was not large enough, (the extent of it is shown upon the models on the table, by a pencil line,) it was increased in size by putting a roll of softened wax to the upper border and moulding it with the finger as a sculptor does his clay, until, after two or three attempts, perfect models of the sides, of sufficient size, were obtained. These were again cast in plaster, and to each was fitted a gold plate; but at this point the real difficulties of the case commenced. It was next necessary that the exact distance which these plates would be from each other when in situ should be ascertained; but many and fruitless were the attempts to secure this, until at last a thin plate of Britannia metal, which could be easily moulded by the finger, was carefully fitted against the surface of one of the plates, to which it was attached by a hinge, so that they might be introduced separately into the mouth, and then united by passing a pin through the hinge. This done, the two side plates were first put into their respective places in the mouth, and then the plate of Britannia metal was introduced and secured on the one side by means of the hinge to the one plate in an exact and known position; the other side was then brought against the other side plate and fitted accurately along its irregular surface by simple pressure of the finger, and for further security the line of the Britannia metal plate was scratched by a fine instrument on the surface of that gold side plate to which it was not attached by the hinge. The three plates were now removed separately from the mouth, the two side plates were placed upon their respective models, and the central plate was secured to the one by the hinge on the one side, and on the other side brought to correspond to the line scratched on the surface of the other gold plate, in which position it was secured by softened wax, while plaster of paris was poured over the whole. In this manner a working model was obtained, upon which the three plates occupied the exact relative position out of the mouth that they were required to occupy in the mouth. This point once attained, it became an easy task to make a model in plaster which should fill up the intervening space between the two side models, resembling as closely as the case would allow an ordinary palate. On this model the centre plate (a copy of which was on the table) was made; and thus, when the three plates were put together, a complete palatine arch was formed.

The method of securing the three plates firmly together when in the mouth is very simple. The two side plates are introduced separately into the mouth first. The right hand plate has a groove running along its surface, into which the corresponding side of the centre plate closely fits. The left hand plate has on its surface one division of a hinge, the other two divisions of which are so secured to the corresponding side of the centre plate that, when it is brought up to its place, which can be accomplished by the tongue, the three divisions of the hinge form one continuous tube, through which is passed a fine gold pin. By this contrivance the three plates are as firmly united when in the mouth, as if they were in one piece. The time required to put the apparatus together in the mouth, or to remove it, does not exceed two minutes. With the plates thus arranged, the voice at once became considerably improved. Many words could be distinctly pronounced; but there was much yet to be desired. It was, therefore, determined to try the effect of a soft palate. Numberless experiments both in material and shape, which it would be useless to describe here, resulted in one, carved in ivory, (a copy of which was laid before the Society,) which restored the voice as completely as without lips it was possible to expect. During these experiments, one fact in reference to the voice came out most strikingly, viz. that there should be no interruption of continuity between the hard and soft palates; even a pin-hole is sufficient to mar the otherwise good effect, as was found again and again. The importance of supplying lips, now for the first time forced itself on my attention. As the disfigurement was very considerable, it was hoped to cover the whole face below the eyes with a mask, if a material could be found flexible and capable of being sharply moulded and receiving paint; vulcanized india-rubber was found to be such a material. A cast of the whole face was taken, and upon it was built up a face by my friend Mr. Durham, the sculptor, in character with the upper part of the head and face, the junction with which it was intended to hide by the help of spectacles. These casts were copied in type metal, and forwarded to Manchester, to Messrs. Macintosh & Co's manufactory, and the india-rubber mask now on the table, was the result. It is defective in several respects, in which a second would have been perfect. Its edges are rough and thick, and there is a hole on one side of the nose, all of which imperfections would have been rectified in a second; but it was not obtained, as our patient expressed an insuperable objection to wearing any kind of mask. All he wished to have, was lips, so that his voice might be perfected. Lips were therefore made, india-rubber being again used for the purpose. A thin gold band with two hooks, was secured to the back of each lip, by which they were to be attached, the upper one to the upper centre plate, and the lower to a plate fitted to the lower jaw, made merely for the purpose of carrying the lip. By this arrangement the lips could be placed and removed at pleasure. With the apparatus thus completed the power of distinct articulation, was, within a week, so far restored that our patient returned to the country to resume duties involving public speaking, from which for years he had been entirely excluded.

The lower lip has been altogether dispensed with, as the plate to which it was attached interfered in some measure with the movements of the soft material covering the chin, which, within the last twelve months, has so gained in fullness and mobility, that there is good reason to hope, that in another year, a useful natural lip will be formed. The material of the upper lip has been changed from india-rubber to ivory, as it was found that the thin edge of the rubber decomposed and softened, and the paint scaled off. The ivory has these advantages over the softer material, that the hooks are fixed more firmly, and also that teeth showing below the lip may be riveted to it. The voice is quite as good with the ivory, as with the india-rubber lip.

The apparatus thus described, has been worn for about nine months, a period long enough to prove it; and it has been found to prevent effectually the passage of fluids from the oral, to the nasal cavities, during the act of deglutition. The sense of taste has been restored, and appears in no measure defective. The sense of hearing, which was completely destroyed in the right ear, and nearly so in the left, is fully restored in the latter; but it remains unimproved in the former. The cause of deafness which the apparatus appears to have removed, in so great a degree was, it would seem, the extension through the Eustachian tubes into the middle ear, of a dried and thickened condition of the mucous membrane of the pharynx, brought on by prolonged and direct exposure to the atmosphere at the ordinary temperature.

The power of articulation is so far restored, that, in a letter recently received, this patient says, "My voice is as good as I could possibly expect, almost as good as I could wish. I am beginning this new year in the greatest hope and confidence of future comfort and usefulness."

From this case, three points appear clearly brought out.

1st. It little matters how long or wide the cleft may be; an obturator can be as easily adapted to the largest as to the smallest opening, therefore, the possibility of an operation for cleft palate being followed by sloughing, such as would enlarge the primary opening, ought not to present any grave objection to its being performed; and consequently, in all cases where there is no contra-indication, the chance of a successful issue should be given.

2d. In cases where the fissure is accidental and not congenital, it requires but that the parts lost, be artificially restored, and almost immediately the power of speech is regained.

3d. That portions of the face which may have been destroyed by accidental causes may be restored by mechanical contrivance when beyond the reach of the surgeon. A fact of no small interest at the present time, when so many of our countrymen are likely to return home variously mutilated.

Appearance of the mouth after perfect cicatrization.

Appearance of the mouth with false lip adjusted.

## Figures and Tables

**Figure f1:**
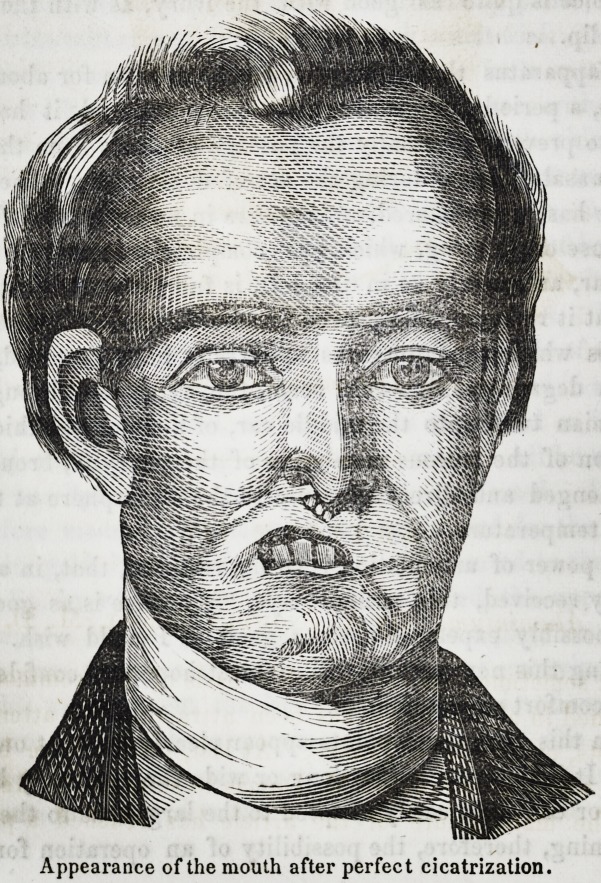


**Figure f2:**